# Prescriptions and Their Owners

**Published:** 1891-05-02

**Authors:** 


### PRESCRIPTIONS AND THEIR OWNERS.

Sir,—The article which appeared in your paper last week on "Prescriptions and their Owners" omits to mention one decided disadvantage of prescription-giving from the patient's side of the question. It is possible the prescription may be the wisest, but just as possible that the chemist to whom it is entrusted may either substitute a wrong drug, or by some other mistake render the medicine futile, if not harmful. It is much more to the patient's interest for the doctor himself to bear the responsibility of preparing the medicine, for then the patient is not left to the mercy of a thoughtless or unscrupulous chemist. From the doctor's point of view I do not quite agree with your article, for it surely requires very little wisdom to understand that the same prescription cannot always be appropriate for the same person even under apparently similar circumstances, and the person who would take medicine prescribed for some one else must be entirely devoid of common sense. I do not think that by giving prescriptions the doctors suffer so much as the patients who are usually victimised by the chemists.—I am, Sir, yours truly,

April 27, 1891. G. N. R.

